# Identity and Personality Pathology in Adult Forensic Psychiatric Patients and Healthy Controls

**DOI:** 10.1177/0306624X241248364

**Published:** 2024-04-23

**Authors:** Deni Tressová, Elien De Caluwé, Stefan Bogaerts

**Affiliations:** 1 Tilburg University, The Netherlands; 2 Fivoor Science and Treatment Innovation, Poortugaal, The Netherlands

**Keywords:** identity, personality disorders, adults, forensic patients, forensic psychiatry

## Abstract

Since the publication of the fifth edition of the *Diagnostic and Statistical Manual of Mental Disorders (DSM-5)*, identity impairment has become a diagnostic criterion for all personality disorders. The current study examined the occurrence of identity dimensions, clinically relevant identity impairments and personality pathology, and associations between these constructs in 92 forensic patients and 139 healthy controls. Patients showed higher levels of almost all identity dimensions, identity impairments, personality disorders, and almost all maladaptive personality traits than controls. Various identity dimensions were associated with consolidated identity as well as identity impairments in both groups. Both patients and controls with high ruminative exploration and identity malfunctioning showed more personality pathology. Different associations between identity functioning and particularly antisocial and borderline personality disorder showed to be stronger in patients than in controls. Our results highlight the importance of identity impairment as a crucial criterion to assess and treat personality pathology in forensic patients.

Most offenders are characterized by personality pathology. The highest prevalence for personality disorders (PDs) is found in adult forensic psychiatric patients (range 80–87%) (Van der Veeken et al., 2018) who mostly suffer from Cluster B PDs. A meta-analysis showed that personality pathology is associated with an increased risk of violent behavior (Yu et al., 2012) and especially the antisocial and narcissistic PDs are associated with criminal behavior and recidivism (Hare, 2006; Howard et al., 2014; Yu et al., 2012). Personality pathology has thus become a crucial aspect of assessment and treatment in forensic psychiatry due to its strong association with criminal behavior and recidivism in various groups of forensic patients (S. Bogaerts, Spreen, et al., 2018). Additionally, identity, particularly the narrative script, has become a key topic in the desistance literature (Johnson & Manura, 2019). According to Maruna (2001), ex-offenders require a coherent self-narrative to explain their transition to reformed identities and maintain abstinence from crime. This new life script offers values that support a crime-free lifestyle despite past adversities (Marsh, 2011). While research has separately explored identity and personality pathology in relation to recidivism and desistance, there remains a need to integrate these fields to uncover new insights for forensic practice.

## Personality Pathology

Since the 5th edition of the *Diagnostic and Statistical Manual of Mental Disorders* (*DSM-5;* American Psychiatric Association [APA], 2013), an alternative model for PDs (AMPD) has been published in Section III. It addresses the limitations of the traditional categorical model of PDs as the latter came under considerable critical attack (e.g., Clark, 2007). The AMPD, in contrast, offers a more dimensional approach to the assessment of PDs, defining them in terms of impairments in personality functioning (criterion A), as well as maladaptive personality traits (criterion B), both evidencing unique predictive validity (Roche, 2018). In criterion A, PDs are described by four domains of personality (mal)functioning: identity and self-direction related to the self, and empathy and intimacy related to interpersonal relationships. In criterion B, personality pathology is defined by five personality trait domains: negative affectivity, detachment, antagonism, disinhibition, and psychoticism. These domains are specified by 25 maladaptive personality traits (MPTs), such as anhedonia, emotional lability, and callousness. Specific combinations of some of these MPTs compose the six PDs from the AMPD perspective (i.e., antisocial, narcissistic, borderline, schizotypal, obsessive-compulsive, and avoidant).

According to the AMPD, identity has now become an important aspect of PDs (Pincus et al., 2020). Identity impairment, namely the pattern of impairments in an individual’s identity, is considered as one of the indicators to diagnose personality pathology. There is sufficient empirical evidence for the association between identity impairment and PDs in adolescents (Westen et al., 2011), community (Bogaerts et al., 2021) and clinical samples (Amini et al, 2015; Bogaerts et al., 2023; Morey et al., 2011). These studies have shown that identity disturbance appears to be an important construct when it comes to borderline, antisocial, narcissistic and schizotypal PD. However, as far as we know, the research on identity and its association with personality pathology in adult *forensic* psychiatric patients is lacking.

## Identity (Mal)Functioning

### Dimensional Identity Perspective

Identity can be conceptualized from a dimensional and a clinical perspective. First, concerning the *dimensional* perspective, a process-oriented model was developed (Luyckx et al., 2006) to understand the underlying processes of identity formation. It distinguishes five dimensions: *exploration in breadth* (looking for new commitments), *exploration in depth* (evaluating current commitments), *ruminative exploration* (endlessly worrying over what choice to make in life), *commitment making* (the degree to which commitments are made in a certain developmental domain), and *identification with commitment* (the degree to which commitments contribute to a sense of self and provide certainty in life).

Identity dimensions are related to both healthy and negative outcomes, such as criminal behavior and aggression (Mercer et al., 2017; Morsunbul, 2015). Particularly ruminative exploration is dysfunctional because it is associated with depression and aggression, hence characterizing the dark side of identity formation (Beyers & Luyckx, 2016; Luyckx et al., 2006; Morsunbul, 2015; Schwartz et al., 2009). This identity dimension is the only one that is negatively associated with the identity dimensions commitment making and identification with commitment (Beyers & Luyckx, 2016; Luyckx et al., 2013). Juvenile delinquents show lower levels of commitment than clinically referred youth and youth from the general population (Klimstra et al., 2011). Adolescents with low levels of commitment report more delinquency than those with high levels of commitment (Meeus et al., 2012). Furthermore, although exploration in breadth and exploration in depth are generally seen as adaptive characteristics of identity formation (Zimmerman et al., 2015), they are both associated with maladjustment indicators like substance use (Luyckx et al., 2006) and aggression during adolescence and early adulthood (Morsunbul, 2015). Thus, high levels of ruminative exploration, exploration in breadth and exploration in depth, and low levels of the two commitment dimensions are associated with maladjustment.

### Clinical Identity Perspective

Second, *clinical* identity functioning can be described in terms of consolidated identity, disturbed identity and lack of identity (Kaufman et al., 2014). Individuals who achieved identity synthesis (or *consolidated identity*), have succeeded in developing commitments and a long-lasting set of values, beliefs and attitudes (Schwartz et al., 2009). Individuals with a *disturbed identity* experience a lack of purpose, direction in life, and commitments to goals, values, and relationships with others. They have a sense of incoherence because they cannot integrate the concept of self and others. Finally, individuals characterized by a *lack of identity* face extreme identity impairments and feelings of non-existence and inner emptiness (Kernberg, 2006).

Individuals with high levels of consolidated identity and low levels of disturbed identity and lack of identity generally report low levels of depression, anxiety and borderline PD (Bogaerts, Claes, et al., 2018; Bogaerts et al., 2023; Kaufman et al., 2015) and other PDs (antisocial, histrionic, dependent, avoidant, schizotypal, paranoid) (Bogaerts et al., 2023). Individuals with disturbances in identity functioning have many problems perceiving themselves as unique, have blurred boundaries between self and others, and have difficulty with emotion regulation. Furthermore, delinquency and externalizing problems are negatively associated with identity synthesis and positively associated with identity confusion (Schwartz et al., 2009). In addition, identity malfunctioning is also related to other negative outcomes, such as criminal behavior (Klimstra et al., 2011; Meeus et al., 2012). Individuals with a maladaptive identity are more likely to persist in committing crimes (Maruna, 2001). Nevertheless, it is notable that research on specifically clinical identity disturbance in psychiatric patients is very limited (Bogaerts et al., 2023; Kaufman et al., 2015; Westen et al., 2011), and absent in forensic patients.

Finally, concerning the interrelations between the two identity perspectives, individuals high on disturbed identity and lack of identity often report low levels of identification with commitment, while consolidated identity is positively linked to exploration in depth and both commitment dimensions, and low levels of ruminative exploration (Bogaerts, Claes, et al., 2018).

## The Current Study

Despite considerable evidence for the role of identity in the development of personality pathology within clinical adult samples (e.g., Amini et al., 2015; Bogaerts et al., 2023), research on the role of identity within personality pathology in adult forensic psychiatric patients is lacking (Billen et al., 2022). Given (a) the high prevalence of PDs among adult forensic psychiatric patients, (b) the importance of identity in diagnosing PDs, and (c) the association of PDs as well as identity with criminal behavior, research on identity and personality pathology in adult forensic patients may provide insights into its relationship with crime and recidivism. Therefore, the current study will focus on adult forensic psychiatric patients and investigate identity functioning and personality pathology, in comparison with healthy controls. Our study contributes to the existing literature by investigating objectives that have not been previously investigated in adult forensic psychiatric patients. We aim to (1) study group differences between forensic patients and controls regarding identity and personality pathology, (2) study the associations between two identity perspectives (i.e., dimensional and clinical), (3) investigate the associations between identity and personality pathology, and (4) determine whether these associations are stronger in forensic patients versus controls (using moderations).

More specifically, first, we will investigate mean level group differences between forensic patients and healthy controls in terms of the presence and level of identity dimensions, clinical identity, and personality pathology—both in a broad (i.e., the PDs) and more specific way (i.e., the MPTs). Existing literature suggested that increased ruminative exploration, exploration in breadth and exploration in depth, and decreased commitment levels are associated with maladjustment. Hence, from a *dimensional* identity perspective, we expect forensic patients to show higher levels of all three exploration dimensions and lower levels of both commitment dimensions than controls (Klimstra et al, 2011; Meeus et al., 2012; Schwartz et al., 2009) [Hypothesis 1a]. From a *clinical* identity perspective, we expect higher levels of disturbed identity and lack of identity, and lower levels of consolidated identity in patients than controls (Bogaerts, Claes, et al., 2018; Kaufman et al., 2015; Schwartz et al., 2009) [Hypothesis 1b]. With respect to personality pathology, we expect that patients compared to controls will report higher levels of all PDs and MPTs, but especially antisocial (manipulativeness, callousness, deceitfulness, hostility, risk taking, impulsivity and irresponsibility), narcissistic (grandiosity and attention seeking), and borderline PD (emotional lability, anxiousness, separation insecurity, depressivity, impulsivity, risk taking, and hostility), as these are most prevalent in forensic patients (De Ruiter & Greeven, 2000; Van der Veeken et al., 2018) [Hypothesis 1c].

Second, we will investigate the link between the two identity perspectives themselves, (i.e., between identity dimensions and clinical identity structures) given the importance of connecting more traditional developmental literature with a clinical identity measure. Based on Bogaerts, Claes, et al. (2018), we expect that high levels of two exploration dimensions (in breadth and depth) and both commitment dimensions as well as low levels of ruminative exploration are linked to high levels of consolidated identity, especially in forensic patients [Hypothesis 2a]. According to Bogaerts, Claes, et al. (2018), identification with commitment is expected to be negatively associated with disturbed identity and lack of identity, particularly in forensic patients [Hypothesis 2b]. Hence, we predicted stronger associations in forensic patients versus controls due to assumed greater identity malfunctioning (see aim 1).

Third, we will investigate the associations between the dimensional and clinical identity approach on the one hand and personality pathology (PDs and their specific MPTs) on the other. From a *dimensional* perspective, given previous research (e.g., Amini et al., 2015), we expect high levels of ruminative exploration and low levels of both commitment dimensions to be positively linked to antisocial, narcissistic, and borderline PD (and their respective MPTs) in both groups [Hypothesis 3a]. From a *clinical* identity perspective, we expect high levels of disturbed identity and a lack of identity, and low levels of consolidated identity to be positively associated with these previously mentioned PDs and MPTs in both groups (Bogaerts, Claes, et al., 2018; Bogaerts et al., 2023; Kaufman et al., 2015, Schwartz et al., 2009) [Hypothesis 3b].

Finally, we expect associations between identity and personality pathology to be stronger in forensic patients compared to controls (tested with moderations) [Hypothesis 4], because we assume that patients will show more identity malfunctioning and personality pathology (see aim 1) and that the association between these constructs will be stronger in patients. This can offer additional support for using identity impairment as one of the key constructs to diagnose PDs.

## Method

### Participants and Procedure

#### Patient Sample

Data collection took place in three forensic psychiatric centers (FPCs) in the Netherlands (Rotterdam) and Belgium (Ghent and Antwerp). Ninety-two male patients participated. See Table S1 (Supplemental Material) for sociodemographic characteristics. Dutch patients received TBS orders for crimes with a minimum sentence of 4 years (“terbeschikkingstelling” in Dutch). TBS entails detention under hospital order with compulsory psychiatric treatment, imposed by court for serious crimes because of severe mental illness. Belgian patients were partially or fully unaccountable due to mental illness when there is a recidivism risk (Van Marle, 2002). Patients residing in Dutch and Flemish FPCs were compared on the 14 items of the Historisch Klinisch Toekomst risk assessment instrument (HKT-R, Spreen et al., 2014) that measures the risk of future violent recidivism in forensic psychiatric patients. There was no significant difference on the clinical total score [*t*(72) = −0.03, *p* = .97] for Dutch (*M* = 1.57, *SD* = 0.95) and Flemish patients (*M* = 1.47, *SD* = 1.21) as well as on the 14 clinical items, except for the item social skills [*t*(73) = −2.15, *p* = .04] where the Dutch patients scored lower (*M* = 1.61, *SD* = 0.78) than the Flemish patients (*M* = 2.13, *SD* = 1.05). Therefore, the Dutch and Flemish patients were combined into one group. Of them, 72% were diagnosed with a PD, whereof 11% were diagnosed with two or three PDs (see Table S1). Other psychiatric diagnoses included substance use disorder, paraphilic disorder, developmental disorder, psychotic disorder, mood disorder, and disruptive disorder. Prior to data collection, all patients were informed about the study. Two weeks later, they were invited to participate, to complete an informed consent and to give permission to use electronic patient data (diagnoses and index crimes). They could stop their participation at any time, without giving any reason and without any consequences. They received a reward of €10 after each assessment.

#### Non-Clinical Control Sample

A sample of 185 men was recruited from the general population in the Netherlands by bachelor and master psychology students at Tilburg University. They were instructed to look for male participants between 25 and 65 years old, with primary or secondary level of education to match the socio-demographic characteristics of the patients in the study as much as possible. Participants were given a hyperlink to the online questionnaires. They were informed about the study and completed an informed consent prior to assessment. Participants received no reward. To ensure the non-clinical nature of this sample, all participants completed the Symptom Checklist (SCL-90; Arrindell & Ettema, 2003) to control for various symptoms of psychopathology. A SCL-90 total score lower than 224 was required for inclusion in the non-clinical sample. Three participants scored higher and were removed. Forty-three participants reported a history of mental health care or imprisonment and were therefore excluded from the study, ultimately resulting in 139 non-clinical controls.

#### Total Sample

The total sample consisted of 231 men (92 patients and 139 controls) with a mean age of 43.9 years. See Table S1 for sociodemographic characteristics and group differences. Most of them were single, European and had secondary education. Compared to the control group, patients were significantly younger [age patients 41.6 (*SD* = 10.3; range = 25–64); age controls 45.4 (*SD* = 13.2; range = 20–70); *t*(218.9) = −2.38; *p* *<* .05], less educated [χ^2^(5) = 68.75; *p* *<* .01] and more single [χ^2^(4) = 96.86; *p* *<* .01]. There were no differences for ethnicity [χ^2^(1) = 0.04; *p* > .05]. Ethical approval was obtained from the Ethics Review Board of Tilburg University (EC-2017.45).

### Measures

#### The Dimensions of Identity Development Scale (DIDS)

The DIDS (Luyckx, Schwartz, et al., 2008) measures five dimensions (exploration in breadth, exploration in depth, ruminative exploration, commitment making, and identification with commitment) by a 5-point Likert-type scale ranging from 1 (*completely untrue*) to 5 (*completely true*). The intercorrelations between the five dimensions are reported in Table S2. Research in community (Luyckx, Schwartz, et al., 2008) and clinical (Bogaerts et al., 2023) samples showed good validity and reliability. Table 1 reports Cronbach’s alpha values of all measures.

**Table 1.** Group Differences Between the Patient and Non-Clinical Sample on the DIDS, SCIM, and PID-5-SF.

	Patients (*n* = 92)			Controls (*n* = 139)			*U*
	*M*	*SD*	α	*M*	*SD*	α	
DIDS							
Exploration in breadth	3.82	0.82	.87	3.36	0.74	.83	4149.500***
Exploration in depth	3.51	0.82	.77	3.12	0.75	.84	4528.000***
Ruminative exploration	2.79	1.08	.85	2.14	0.82	.88	4137.000***
Commitment making	4.12	0.85	.93	3.75	0.76	.87	4398.000***
Identification with commitment	3.88	0.81	.87	3.65	0.65	.87	4981.000**
SCIM							
Consolidated identity	5.18	0.92	.68	5.43	0.61	.54	5513.000
Disturbed identity	3.07	1.18	.84	2.22	0.77	.85	3573.500***
Lack of identity	3.03	1.32	.82	1.69	0.75	.84	2440.000***
PDs							
Antisocial PD	0.94	56	.94	0.66	0.37	.91	4400.000***
Avoidant PD	0.87	0.56	.88	0.45	0.34	.83	3517.500***
Borderline PD	1.08	0.61	.93	0.62	0.33	.87	3364.500***
Narcissistic PD	0.94	0.60	.81	0.69	0.50	.84	4874.500**
Obsessive-compulsive PD	1.04	0.56	.88	0.73	0.40	.84	4158.000***
Schizotypal PD	0.91	0.53	.91	0.67	0.31	.88	4544.000***
MPTs							
Attention seeking	1.08	0.75	.81	0.85	0.63	.83	5330.500*
Callousness	0.71	0.69	.80	0.40	0.46	.78	4823.000**
Deceitfulness	0.76	0.68	.75	0.51	0.49	.73	5072.500**
Grandiosity	0.80	0.68	.73	0.54	0.49	.69	4964.500**
Hostility	1.05	0.75	.82	0.77	0.57	.78	4970.000**
Manipulativeness	0.92	0.70	.79	0.94	0.65	.83	6140.500
Anhedonia	0.77	0.64	.74	0.31	0.36	.64	3610.500***
Depressivity	0.71	0.65	.70	0.13	0.26	.76	2643.500***
Intimacy avoidance	0.67	0.68	.76	0.44	0.50	.67	5242.000*
Restricted affectivity	1.08	0.71	.73	0.92	0.58	.73	5664.500
Withdrawal	0.95	0.70	.73	0.48	0.46	.68	3792.000***
Anxiousness	1.07	0.80	.82	0.57	0.50	.75	4071.500***
Emotional lability	1.07	0.76	.79	0.54	0.45	.66	3767.000***
Perseveration	1.06	0.73	.83	0.70	0.47	.67	4518.000***
Separation insecurity	1.28	0.82	.77	0.73	0.55	.67	3899.500***
Submissiveness	1.00	0.62	.69	0.84	0.56	.78	5439.500
Suspiciousness	1.15	0.66	.74	0.53	0.41	.56	2842.500***
Distractibility	1.11	0.84	.88	0.88	0.74	.90	5386.000*
Impulsivity	1.21	0.67	.69	0.87	0.50	.63	4428.500***
Irresponsibility	0.77	0.63	.69	0.41	0.40	.55	4221.500***
Risk taking	1.19	0.82	.83	0.72	0.56	.78	4303.500***
Rigid perfectionism	1.34	0.73	.76	0.84	0.63	.80	3834.500***
Eccentricity	0.94	0.72	.81	1.66	0.54	.84	2977.500***
Perceptual dysregulation	0.57	0.69	.82	0.16	0.28	.70	4232.500***
Unusual beliefs	0.78	0.66	.62	0.24	0.36	.60	3197.500***

*Note*. α = Cronbach’s alpha values.

* *p* < .05. **p < .01. ***p < .001.

#### The Self-Concept and Identity Measure (SCIM)

The SCIM (Kaufman et al., 2015) measures identity consolidation and clinically relevant identity disturbance. It includes three scales: consolidated identity, disturbed identity, and lack of identity, rated on a 7-point Likert-type scale ranging from 1 (*completely disagree*) to 7 (*completely agree*). The intercorrelations between the three scales are as follows: consolidated identity and disturbed identity (*r* = 0.09, *p* = ns in patients; *r* = −0.15, *p* = ns in controls), consolidated identity and lack of identity (*r* = 0.08, *p* = ns in patients; *r* = −0.25, *p* < .008 in controls), and disturbed identity and lack of identity (*r* = 0.59, *p* < .001 in patients; *r* = 0.64, *p* < .001 in controls) (see Table S2). The SCIM is reliable and structurally valid in community (Bogaerts et al., 2021; Kaufman et al., 2015) and clinical samples (Bogaerts et al., 2023; Kaufman et al., 2019).

#### Personality Inventory for DSM-5 Short Form (PID-5-SF)

The PID-5-SF (Maples et al., 2015) is a 100-item self-report scale that measures *DSM-5* MPTs. It comprises 25 lower order MPTs measured by a 4-point Likert-type scale ranging from 0 (*very false or often false*) to 3 (*often true or very true*). To calculate the six PDs (i.e., antisocial, avoidant, borderline, narcissistic, obsessive-compulsive and schizotypal PD), the mean score of their respective MPTs was calculated (APA, 2013). The PID-5-SF shows a comparable factor structure, validity and reliability as the original PID-5, which are all adequate across community and clinical participants (e.g., De Caluwé et al., 2019; for a review, see Al-Dajani et al., 2016) as well as incarcerated offenders (Dunne et al., 2021). Further psychometric research in forensic samples is needed (Hopwood & Sellbom, 2013) and the current study adds to this.

#### Symptom Checklist-90-Revised (SCL-90-R)

The SCL-90-R (Arrindell & Ettema, 2003) was used in the control group to check for general psychological distress. All 90 items were rated on a 5-point Likert-type scale going from 1 (*not at all*) to 5 (*extremely*). It showed sufficient psychometric properties (Bech et al., 2014) and an excellent Cronbach’s alpha value of .95 in the current non-clinical control sample.

### Statistical Analyses

An a priori power analysis using G*Power 3.1 (Faul et al., 2007) with a medium effect size (*f*^2^ = 0.15), an alpha of .05 and up to eight predictors (the scales of DIDS or SCIM, age, education and group as moderator) showed that 231 participants were sufficient to achieve a power of .95. Because of violated assumptions, non-parametric analyses were conducted. Mann-Whitney *U* tests were used to test group differences (aim 1). Spearman’s Rho correlations were computed to investigate the associations among these constructs in both samples, adopting Bonferroni corrections (aims 2 and 3). To test whether belonging to the patient/control sample moderated the relationships between DIDS/SCIM and PID-5-SF, moderation analyses were performed using PROCESS (Hayes, 2013), allowing for a non-parametric approach (bootstrapping) (aim 4). The independent variables (DIDS and SCIM scores) and the moderator were centered. Because of the violation of the assumption of normal distribution, effect estimates were based on biased-corrected 95% bootstrap confidence intervals with 1,000 bootstrap resamples. To reveal the unique effects of the identity scales, we controlled for the other scales, age and education level in the moderations. To calculate the effect sizes, the *R*^2^ values were converted into *f*^2^ values. The effect sizes were interpreted according to Cohen (1988) where *f*^2^ < 0.02 is seen as small, 0.15 as medium, and 0.35 as large.

## Results

### Mann-Whitney U Group Differences

Table 1 shows that patients reported significantly higher scores on all five DIDS scales and two SCIM scales (disturbed identity and lack of identity). Patients also scored significantly higher than controls on all PID-5-SF PDs and on almost all MPTs, except for eccentricity where patients scored significantly lower. The greatest group differences were observed in depressivity, suspiciousness, and eccentricity.

### Spearman’s Rho Correlational Analyses in Subgroups

Spearman’s Rho correlations between the DIDS, SCIM and PID-5-SF PDs are in Table S2, and between the DIDS, SCIM and PID-5-SF MPTs in Table S3, per subgroup. First, we investigated associations between identity dimensions (DIDS) and clinical identity structures (SCIM). Exploration in breadth (*r* = 0.40, *p* < .001) and commitment making (*r* = 0.34, *p* < .008) were positively related to consolidated identity in patients, while in controls, exploration in breadth showed a positive association with disturbed identity (*r* = 0.25, *p* < .008). In both samples, ruminative exploration showed positive associations with disturbed identity (*r* = 0.33, *p* < .008 in patients; *r* = 0.33, *p* < .001 in controls) and lack of identity (*r* = 0.39, *p* < .001 in patients; *r* = 0.48, *p* < .001 in controls). In both samples, identification with commitment was positively related to consolidated identity (*r* = 0.32, *p* < .008 in patients; *r* = 0.27, *p* < .008 in controls).

Second, regarding the associations between the DIDS and both PID-5-SF PDs (Table S2) and MPTs (Table S3), in both samples, ruminative exploration was positively associated with antisocial PD (*r* = 0.32, *p* < .008 in patients; *r* = 0.22, *p* < .008 in controls), avoidant PD (the strongest association; *r* = 0.44, *p* < .001 in patients; *r* = 0.34, *p* < .001 in controls), borderline PD (*r* = 0.40, *p* < .001 in patients; *r* = 0.31, *p* < .001 in controls), and schizotypal PD (*r* = 0.38, *p* < .001 in patients; *r* = 0.30, *p* < .001 in controls) and with the MPTs hostility (*r* = 0.43, *p* < .001 in patients; *r* = 0.28, *p* < .008 in controls), anhedonia (*r* = 0.36, *p* < .001 in patients; *r* = 0.29, *p* < .001 in controls), depressivity (*r* = 0.35, *p* < .001 in patients; *r* = 0.34, *p* < .001 in controls), anxiousness (*r* = 0.39, *p* < .001 in patients; *r* = 0.40, *p* < .001 in controls) and eccentricity (*r* = 0.32, *p* < .001 in patients; *r* = 0.30, *p* < .008 in controls). In addition, in patients, ruminative exploration was also associated with obsessive-compulsive PD (*r* = 0.34, *p* < .008), perseveration (*r* = 0.32, *p* < .008), irresponsibility (*r* = 0.34, *p* < .008) and perceptual dysregulation (*r* = 0.33, *p* < .001), and with unusual beliefs in controls (*r* = 0.30, *p* < .001). Specifically, in the control sample, identification with commitment was negatively associated with avoidant PD (*r* = −0.26, *p* < .008), and exploration in depth was positively associated with manipulativeness (*r* = 0.27, *p* < .008). Finally, commitment making showed a positive association with rigid perfectionism, but only in patients (*r* = 0.32, *p* < .008).

Finally, regarding the associations between the SCIM and both PID-5-SF PDs (Table S2) and MPTs (Table S3), in both samples, disturbed identity showed positive associations (often strong in patients) with all six PDs (all *p* < .001)—antisocial (*r* = 0.55 in patients; *r* = 0.51 in controls), avoidant (*r* = 0.37 in patients; *r* = 0.39 in controls), borderline (*r* = 0.55 in patients; *r* = 0.55 in controls), narcissistic (*r* = 0.57 in patients; *r* = 0.47 in controls), obsessive-compulsive (*r* = 0.45 in patients; *r* = 0.43 in controls) and schizotypal (*r* = 0.55 in patients; *r* = 0.47 in controls)—and almost all MPTs (*r*s ranging from 0.32, *p* < .008 [anhedonia] to 0.61, *p* < .001 [hostility] in patients; and from 0.26, *p* < .008 [depressivity] to 0.50, *p* < .001 [deceitfulness] in controls), except for restricted affectivity, withdrawal and rigid perfectionism in patients, and callousness and intimacy avoidance in controls. Lack of identity was positively and the most strongly associated with all PDs in patients (all *p* < .001)—antisocial (*r* = 0.57), avoidant (*r* = 0.62), borderline (*r* = 0.73), narcissistic (*r* = 0.43), obsessive-compulsive (*r* = 0.52) and schizotypal PD (*r* = 0.64)—and in controls (all *p* < .001) with avoidant (*r* = 0.42), borderline (*r* = 0.34), obsessive-compulsive (*r* = 0.30), and schizotypal PD (*r* = 0.40). Further, lack of identity was positively associated with almost all MPTs (*r*s ranging from 0.32, *p* < .008 [callousness] to 0.62, *p* < .001 [hostility] in patients; and from 0.27, *p* < .008 [callousness] to 0.43, *p* < .001 [depressivity] in controls), except for attention seeking, restricted affectivity and submissiveness in patients, and attention seeking, hostility, manipulativeness, intimacy avoidance, perseveration, distractibility, impulsivity, risk taking, and rigid perfectionism in controls. Finally, consolidated identity was not significantly related to any of the PDs or MPTs in both groups.

### Moderation Analyses

All models predicting PID-5-SF PDs (see Table 2) and MPTs^1^ were significant, except when restricted affectivity was predicted by exploration in depth, and restricted affectivity and submissiveness were predicted by the other four DIDS scales.

**Table 2.** Bootstrap Results of Moderation Analyses for DIDS and SCIM as Predictor of PID-5-SF PDs and Group as Moderator.

	Antisocial PD	Avoidant PD	Borderline PD	Narcissistic PD	Obsessive–compulsive PD	Schizotypal PD
DIDS						
Exploration in breadth	*R*^2^ = .19***	*R*^2^ = .31***	*R*^2^ = .32***	*R*^2^ = .11**	*R*^2^ = .17***	*R*^2^ = .21***
IA	−.08 (−.25, .09)	.01 (−.15, .14)	−.08 (−.27, .09)	−.01 (−.27, .19)	−.01 (−.20, .17)	−.02 (−.18, .14)
PRED	.15 (−.16, .44)	−.08 (−.44, .22)	.09 (−.23, .45)	.08 (−.24, .52)	−.04 (−.35, .32)	.03 (−.26, .33)
MOD	.14 (−.46, .76)	−.33 (−.83, .26)	−.01 (−.65, .68)	−.09 (−.84, .83)	−.21(−.79, .52)	−.04 (−.60, .62)
Exploration in depth	*R*^2^ = .19***	*R*^2^ = .31***	*R*^2^ = .32***	*R*^2^ = .11**	*R*^2^ = .17***	*R*^2^ = .21***
IA	−.03 (−.19, .13)	.002 (−.16, .12)	−.09 (−.25, .07)	−.07 (−.28, .10)	−.02 (−.22, .13)	−.06 (−.21, .08)
PRED	−.04 (−.15, .08)	−.05 (−.14, .06)	−.004 (−.11, .10)	−.04 (−.16, .10)	−.08 (−.19, .06)	−.07 (−.16, .02)
MOD	−.14 (−.30, .01)	**−.29 (−.41, −.15)**	**−.31 (−.47, −.16)**	−.12 (−.19, .06)	**−.22 (−.37, −.06)**	−.09 (−.23, .05)
Ruminative exploration	*R*^2^ = .19***	*R*^2^ = .32***	*R*^2^ = .32***	*R*^2^ = .11**	*R*^2^ = .17***	*R*^2^ = .21***
IA	−.06 (−.20, .07)	−.06 (−.17, .06)	−.07 (−.18, .05)	.07 (−.10, .23)	−.06 (−.18, .07)	−.05 (−.17, .06)
PRED	**.13 (.06, .20)**	**.20 (.14, .27)**	**.18 (.10, .24)**	**.11 (.02, .21)**	**.15 (.08, .22)**	**.17 (.11, .24)**
MOD	**−.14 (−.29, −.01)**	**−.29 (−.42, −.15)**	**−.32 (−.46, −.17)**	−.13 (−.31, .04)	**−.22 (−.36, −.07)**	−.09 (−.22, .04)
Commitment making	*R*^2^ = .19***	*R*^2^ = .31***	*R*^2^ = .32***	*R*^2^ = .12***	*R*^2^ = .18***	*R*^2^ = .21***
IA	−.08 (−.23, .05)	−.01 (−.14, .12)	−.09 (−.24, .05)	−.18 (−.39, .01)	−.11 (−.26, .03)	−.06 (−.19, .08)
PRED	**.13 (.01, .24)**	.11 (−.01, .23)	**.17 (.06, .29)**	.09 (−.05, .22)	.10 (−.01, .22)	**.11 (.01, .22)**
MOD	−.14 (−.28, .01)	**−.29 (−.42, −.16)**	**−.31 (−.47, −.16)**	−.11 (−.28, .08)	**−.21 (−.35, −.07)**	−.09 (−.21, .03)
Identification with commitment	*R*^2^ = .19***	*R*^2^ = .31***	*R*^2^ = .32***	*R*^2^ = .34**	*R*^2^ = .18***	*R*^2^ = .21***
IA	−.06 (−.23, .11)	−.04 (−.22, .13)	−.05 (−.24, .11)	−.15 (−.37, .05)	−.13 (−.32, .04)	−.09 (−.26, .04)
PRED	−.08 (−.21, .04)	−.05 (−.15, .06)	**−.13 (−.25, −.01)**	−.05 (−.20, .09)	.02 (−.09, .13)	−.01 (−.11, .09)
MOD	−.14 (−.29, .0001)	**−.29 (−.42, −.15)**	**−.32 (−.47, −.17)**	−.12 (−.30, .05)	**−.21 (−.35, −.06)**	−.09 (−.22, .05)
SCIM						
Consolidated identity	*R*^2^ = .41***	*R*^2^ = .45***	*R*^2^ = .54***	*R*^2^ = .38***	*R*^2^ = .30***	*R*^2^ = .42***
IA	.03 (−.10, .16)	.11 (−.02, .23)	.08 (−.05, .21)	.002 (−.18, .18)	.04 (−.12, .20)	.04 (−.10, .16)
PRED	**.08 (.0001, .15)**	−.02 (−.09, .05)	.05 (−.02, .12)	**.10 (.01, .19)**	.07 (−.02, .14)	.05 (−.03, .12)
MOD	.02 (−.12, .14)	−.05 (−.17, .08)	−.07 (−.19, .04)	.03 (−.12, .19)	−.05 (−.22, .09)	.09 (−.02, .20)
Disturbed identity	*R*^2^ = .41***	*R*^2^ = .45***	*R*^2^ = .54***	*R*^2^ = .38***	*R*^2^ = .30***	*R*^2^ = .42***
IA	−.02 (−.12, .10)	−.02 (−.13, .11)	−.02 (−.12, .08)	−.01 (−.15, .11)	.004 (−.13, .13)	−.04 (−.14, .06)
PRED	**.20 (.13, .28)**	.03 (−.03, .10)	**.14 (.07, .22)**	**.30 (.21, .38)**	**.12 (.05, .21)**	**.11 (.06, .18)**
MOD	.01 (−.12, .15)	−.06 (−.18, .07)	−.08 (−.20, .04)	.03 (−.14, .17)	−.06 (−.21, .10)	.08 (−.03, .20)
Lack of identity	*R*^2^ = .42***	*R*^2^ = .45***	*R*^2^ = .57***	*R*^2^ = .38***	*R*^2^ = .31***	*R*^2^ = .43***
IA	**−.13 (−.23, −.01)**	−.07 (−.18, .05)	**−.18 (−.29, −.07)**	−.06 (−.20, .10)	−.08 (−.20, .05)	−.08 (−.17, .03)
PRED	.05 (−.01, .13)	**.20 (.13, .28)**	.14 (.08, .21)	.004 (−.08, .10)	**.10 (.02, .19)**	**.13 (.08, .21)**
MOD	.01 (−.11, .13)	−.07 (−.19, .07)	−.09 (−.20, .02)	.03 (−.13, .18)	−.06 (−.20, .10)	.08 (−.03, .20)

*Note.* Statistically significant results are presented in bold. Patient group = 1, control group = 0. Results of covariates are not included, but available upon request. LLCI = lower limit confidence interval; ULCI = upper limit confidence interval; IA = interaction between predictor x moderator, PRED = main effect predictor; MOD = main effect moderator group.

* *p* < .05. **p < .01. ***p < .001.

#### DIDS

Commitment making showed an interaction effect with group in predicting attention seeking, deceitfulness and rigid perfectionism. More specifically, among patients, but not controls, a higher score on commitment making was predictive of higher scores on the PID-5-SF MPTs attention seeking (patients: *b* = 0.29 [0.07, 0.51], controls: *b* = 0.06 [−0.14, 0.27]), deceitfulness (patients: *b* = 0.25 [0.06, 0.45], controls: *b* = 0.08 [−0.09, 0.25]), and rigid perfectionism (patients: *b* = 0.34 [0.12, 0.55], controls: *b* = 0.10 [−0.10, 0.31]). Overall, the effect sizes were medium to large (*f*^2^ range = 0.12–0.47) (Cohen, 1988). Furthermore, exploration in breadth, exploration in depth, ruminative exploration, and identification with commitment showed no interaction effects between groups and any outcome variable, so main effects of the identity scales were interpreted.

More exploration in breadth significantly predicted more hostility. Exploration in depth negatively predicted the MPTs callousness and withdrawal. Ruminative exploration positively predicted all PID-5-SF PDs (especially the avoidant and borderline PDs) and all MPTs, except for attention seeking, manipulativeness, restricted affectivity, distractibility and risk taking. Commitment making positively predicted antisocial, borderline, and schizotypal PD, as well as the MPTs anxiousness, emotional lability, perseveration, separation insecurity, impulsivity, risk taking, perceptual dysregulation, and unusual beliefs. Finally, identification with commitment negatively predicted borderline PD, as well as the MPTs emotional lability, separation insecurity, suspiciousness, distractibility, and impulsivity.

#### SCIM

Consolidated identity, disturbed identity and a lack of identity showed interaction effects with group in predicting several PID-5-PDs and MPTs. More specifically, only in patients, more consolidated identity was predictive of less anhedonia (patients: *b* =* −*0.12 [−0.21, −0.03], controls: *b* = 0.06 [−0.06, 0.17]). Furthermore, in patients but not in the control group, a higher score on disturbed identity was predictive of higher scores on perceptual dysregulation (patients: *b* = 0.25 [0.17, 0.34], controls: *b* = 0.07 [−0.02, 0.17]). Depressivity did not show significant results regarding a specific group (patients: *b* = 0.07 [−0.01, 0.15], controls: *b* =* −*0.06 [−0.15, 0.03]), although the interaction effect was significant and the *b* values in the two groups differ in direction. Further, having a higher score on lack of identity significantly predicted a higher score on depressivity in both groups, and especially in patients (patients: *b* = 0.30 [0.24, 0.37], controls: *b* = 0.17 [0.08, 0.26]). Finally, only in patients, having a higher score on lack of identity significantly predicted a higher score on antisocial PD (patients: *b* = 0.13 [0.06, 0.19], controls: *b* = 0.001 [−0.09, 0.09]) and borderline PD (patients: *b* = 0.25 [0.19, 0.32], controls: *b* = 0.07 [−0.01, 0.16]), as well as on hostility (patients: *b* = 0.20 [0.10, 0.30], controls: *b* = 0.02 [−0.12, 0.15]), perseveration (patients: *b* = 0.17 [0.08, 0.26], controls: *b* =* −*0.02 [−0.15, 0.11]), impulsivity (patients: *b* = 0.19 [0.09, 0.28], controls: *b* =* −*0.04 [−0.17, 0.08]), risk taking (patients: *b* = 0.24 [0.14, 0.35], controls: *b* =* −*0.06 [−0.21, 0.09]), perceptual dysregulation (patients: *b* = 0.21 [0.14, 0.28], controls: *b* = 0.01 [−0.08, 0.11]), and unusual beliefs (patients: *b* = 0.17 [0.09, 0.25], controls: *b* = 0.02 [−0.09, 0.13]). Overall, according to Cohen (1988), the effect sizes were large (*f*^2^ range = 0.43–1.33). Regarding the main effects, consolidated identity positively predicted antisocial and narcissistic PD, as well as the MPTs attention seeking, suspiciousness, distractibility and risk taking. Disturbed identity positively predicted all PID-5-SF PDs (except for avoidant PD) and all MPTs, except for anhedonia, withdrawal, anxiousness and rigid perfectionism. Finally, lack of identity positively predicted avoidant, obsessive-compulsive and schizotypal PD, as well as anhedonia, intimacy avoidance, restricted affectivity, withdrawal, anxiousness, emotional lability, separation insecurity, suspiciousness, and eccentricity.

## Discussion

This study investigated identity dimensions and clinically relevant identity constructs in adult forensic psychiatric patients and healthy controls, and how these two identity perspectives were related to each other and to personality pathology, also focusing on group differences.

First, the hypothesis regarding the dimensional identity perspective is only partially accepted because forensic patients showed not only higher levels of all three exploration dimensions, but also of both commitment dimensions than the control group. The latter might be explained by social desirability because forensic patients tend to give socially desirable answers in self-report questionnaires (Tan & Grace, 2008). From the perspective of clinical identity, as expected, forensic patients reported higher levels of disturbed identity and lack of identity than controls, and unexpectedly, no significant difference was found for consolidated identity. Further, our findings are broadly consistent with the hypotheses on personality pathology, as patients scored higher than controls on all six PDs and almost all MPTs, with the greatest group differences on borderline PD, as expected. Other group differences were found mainly on avoidant and obsessive-compulsive PD, but not on antisocial and narcissistic PD, although this was expected. In more detail, the groups differed on MPTs, mainly for borderline, schizotypal, and avoidant PD, with patients having elevated MPT scores.

Second, regarding the association between the two identity perspectives, as expected, a positive association was observed between exploration in breadth, commitment making and identification with commitment on the one hand, and consolidated identity on the other. These associations were found only in patients, while unexpectedly a positive association between exploration in breadth and disturbed identity was found in controls. Contrary to our hypotheses, exploration in depth was not positively associated with consolidated identity, and ruminative exploration was not negatively associated with consolidated identity. Ruminative exploration, however, showed positive associations with disturbed identity and lack of identity in both samples. The hypothesized negative associations between identification with commitment on the one hand, and disturbed identity and a lack of identity on the other hand were not accepted. This can be attributed to the correction for multiple testing. The unexpected result regarding a positive link between exploration in breadth and disturbed identity might be explained by previous research focusing on exploration in depth, the second adaptive identity dimension (Zimmerman et al., 2015), in which a ruminative component was found. Our results underline this possibility regarding exploration in breadth, since it showed a positive association with ruminative exploration in the control group. Exploration in breadth may contain a maladaptive component, as it was found to be related to substance use (Luyckx et al., 2006).

Third, regarding the expected associations between identity dimensions and personality pathology, ruminative exploration indeed showed positive associations with antisocial and borderline PD, and their various MPTs in both groups, but not with narcissistic PD or any of its MPTs. Unexpectedly, low levels of commitment dimensions were hardly associated with PDs and MPTs in both groups. As for the expected associations between clinical identity and personality pathology, although consolidated identity yielded no associations with PDs or MPTs, in line with Bogaerts et al. (2023) disturbed identity and a lack of identity, as hypothesized, showed positive associations with all PDs, and almost all MPTs in both groups.

Finally, as expected, the moderation analyses revealed that various associations between the dimensional and clinical identity approach on the one hand, and personality pathology on the other, were stronger in forensic patients than in controls. The positive associations between commitment making and some of the MPTs belonging to antisocial, narcissistic and obsessive-compulsive PD were stronger in patients than in controls. The same counts for identity malfunctioning (lack of identity) and antisocial and borderline PD, two of the most common PDs among forensic patients (Van der Veeken et al., 2018).

This study has several strengths. First, our sample consisted of forensic patients, a challenging group to investigate and recruit. Second, we included a dimensional and clinical identity perspective to investigate different identity dimensions in forensic patients during clinical treatment, and to examine the clinical state of the identity development. Third, we used a multifaceted identity model differentiating between various exploration and commitment dimensions. Thus, this paper provides detailed findings on identity impairment that have not been investigated yet in forensic psychiatric patients. Our results illustrate the importance of using a comprehensive model and including different identity perspectives when investigating identity. Finally, including both PDs and MPTs provided more detailed insights about the associations between identity and personality pathology.

Limitations of the current study concern the cross-sectional research design. Therefore, the direction of effects between identity and PDs could not be explored. In this study, length of stay in the forensic treatment center was not included as a covariate in the analyses, which should be done in future studies. Furthermore, patients were not screened for invalid responding (i.e., social desirability). Both groups consisted exclusively of male participants, so generalization to a (forensic) female population is not possible. A final limitation concerns the validity of the two identity instruments, which have not been validated in forensic samples, only in community (Bogaerts et al., 2021; Kaufman et al., 2015; Luyckx, Schwartz, et al., 2008) and clinical samples (Bogaerts et al., 2023; Kaufman et al., 2019).

### Suggestions for Future Research

Further psychometric research on the DIDS and SCIM in forensic samples is crucial. Our study initiated this, but subsequent research must discern if unexpected results represent clinical relevance or measurement validity issues. In contrast, the PID-5-SF was validated in incarcerated offenders (Dunne et al., 2021). Recently, a forensic-specific tool, the Personality Inventory for DSM-5 Forensic Faceted Brief Form (PID-5-FFBF; Niemeyer, 2022) was introduced for assessing maladaptive personality, suggesting similar adaptations for DIDS and SCIM.

Further, since forensic patients explore more, report more commitments and identify themselves more with them when compared to healthy controls, it is important to investigate the nature of these commitments in future research because they may contain a strong deviant character that could be linked to criminal behavior. Moreover, longitudinal studies should be designed to reveal whether identity predicts personality pathology, and/or the other way around. Furthermore, future research should also include other mental health samples, such as community samples with mental health issues or non-forensic clinical samples or forensic outpatients, to be able to compare these groups.

### Clinical Implications

This study shows that forensic psychiatric patients score higher on identity dimensions as well as clinical identity malfunctioning than healthy individuals. These findings are important for appropriate assessment with sufficient depth regarding identity impairment. According to the Good Lives Model (Ward & Marshall, 2007), offenders, such as all other individuals, want to achieve a meaningful life by fulfilling their commitments. Criminal behavior is seen as a harmful way of meeting the same needs that everyone has. Hence, identity development should be taken into account when assessing forensic patients, especially those with antisocial and borderline PD.

Moreover, since our findings showed higher scores on identity dimensions in forensic psychiatric patients, it is likely that identity plays an important role for patients during their stay in forensic institutions. Evaluation of current commitments that might be related to delinquent behavior (exploration in depth) and thinking about and exploring new possibilities to create a new delict-free lifepath in the future (exploration in breadth), seem to be elements that can be stimulated during treatment. Maruna (2001) suggests that supporting ex-offenders in sharing their stories is essential for grasping their recovery processes and understanding how they create new lives. Furthermore, the current study shows that forensic patients have difficulties, at least more than healthy individuals, to finish the process of exploration and to form new commitments (ruminative exploration). Therefore, the current study advocates the inclusion of identity measures not only during the diagnostic phase, but also during treatment. It could be helpful for clinical practitioners working with this group of patients to assess the current state of a patient at the start of treatment both in terms of identity dimensions and clinical identity. This knowledge can help to identify potential risks regarding delict related commitments, and to tailor it to the treatment related needs of a particular patient. This corresponds with the principles of the well-known and generally used Risk-Need-Responsivity (RNR; Andrews & Bonta, 2010) model, describing important principles in reducing recidivism, where identity can be seen as a non-criminogenic “need” that may reduce the risk of recidivism. Identity impairment is prevalent across all PDs in forensic patients, particularly in borderline and antisocial PDs (resulting from our moderations). Targeting identity dysfunction may benefit all PDs. However, PDs vary in their associations with DIDS and SCIM, impacting identity functioning severity. Identity can thus also serve as the “risk principle” in determining treatment intensity, with more maladaptive identity requiring more intensive treatment. This aligns with findings on AMPD’s clinical use (Bach & Tracy, 2022). Effective treatment approaches prioritize interventions addressing self and interpersonal functioning, such as Mentalization-Based Treatment (MBT), over symptom reduction strategies (Rossouw & Fonagy, 2012). Further, also schema therapy can be used to break through limiting beliefs and patterns and develop a more positive view of life, others, and self. This can strengthen a person’s identity and help them function better in everyday life (Bernstein et al., 2023). In treatment, helping forensic patients in building a self-narrative, as suggested by Maruna (2001), is important for achieving desistance. This includes reflecting on past choices, embracing conventional values, and setting new goals. Engaging in activities like volunteering helps offenders to play a positive role in society and serve as examples for others (Maruna, 2001).Therapy should emphasize new exploration, commitment making, and identification with commitment, fostering a consolidated identity and avoiding disturbances. Combining identity-focused therapy with schema/MBT therapy can have benefits, particularly in forensic patients, integrating these approaches for enhanced forensic practice.

## Conclusion

The present study shows that forensic psychiatric patients experience higher levels of identity dimensions, more identity malfunctioning and more personality pathology when compared to a non-clinical control group. Especially the associations between identity malfunctioning and both antisocial and borderline PD appear to be stronger in forensic patients.

## Supplemental Material

sj-docx-1-ijo-10.1177_0306624X241248364 – Supplemental material for Identity and Personality Pathology in Adult Forensic Psychiatric Patients and Healthy Controls Supplemental material, sj-docx-1-ijo-10.1177_0306624X241248364 for Identity and Personality Pathology in Adult Forensic Psychiatric Patients and Healthy Controls by Deni Tressová, Elien De Caluwé and Stefan Bogaerts in International Journal of Offender Therapy and Comparative Criminology

## Notes

1. Bootstrap results of the moderation analyses for DIDS and SCIM as predictor of PID-5-SF maladaptive personality traits and group as moderator are available upon request.
