# Supplementary material for: Identity and Personality Pathology in Adult Forensic Psychiatric Patients and Healthy Controls
Source: Int J Offender Ther Comp Criminol. 2024 Apr 23;68(15):1558–78. doi: 10.1177/0306624X241248364 (PMC11459868; doi:10.1177/0306624X241248364)
Supplement: sj-docx-1-ijo-10.1177_0306624X241248364 – Supplemental material for Identity and Personality Pathology in Adult Forensic Psychiatric Patients and Healthy Controls [file sj-docx-1-ijo-10.1177_0306624X241248364.docx]

**Supplement**

**Table S1**
*Sociodemographic characteristics*

|  |  | Patients (*n*=92) | Controls (*n*=139) | Group differences |
| --- | --- | --- | --- | --- |
| Age (years) |  | 41.6 (*SD*=10.3;  range=25-64) | 45.4 (*SD*=13.2; range=20-70) | *t*(218.9)=-2.38* |
| Gender (%) | Male | 100 | 100 |  |
| Ethnicity (%) | European | 88 | 89 | *χ2*(1)=.04 |
|  | Non-European | 12 | 11 |  |
| Education (%) | Elementary | 22 | 0 | *χ2*(5)=68.75*** |
|  | Lower | 22 | 22 |  |
|  | Secondary | 35 | 44 |  |
|  | Higher | 3 | 27 |  |
|  | University | 3 | 7 |  |
|  | Other | 15 | 0 |  |
| Marital status (%) | Single | 72 | 30 | *χ2*(4)=96.86*** |
|  | Married | 2 | 65 |  |
|  | Relationship | 11 | 0 |  |
|  | Divorced | 13 | 4 |  |
|  | Widowed | 0 | 1 |  |
|  | Other | 2 | 0 |  |
| Crime (%) | Child sexual abuse | 33 |  |  |
|  | Violence and possession of weapons | 21 |  |  |
|  | Property offences | 19 |  |  |
|  | Homicide | 17 |  |  |
|  | Sexual offences other than child sexual abuse | 16 |  |  |
|  | Physical assault | 14 |  |  |
|  | Murder | 12 |  |  |
|  | Arson | 8 |  |  |
|  | Property offences with violence | 7 |  |  |
|  | Destruction of goods | 4 |  |  |
|  | Traffic offences and public disruption | 4 |  |  |
|  | Drug offences | 1 |  |  |
| PD (%) | Antisocial PD | 41 |  |  |
|  | PD-not otherwise specified | 21 |  |  |
|  | Borderline PD | 13 |  |  |
|  | Narcissistic PD | 6 |  |  |
|  | Avoidant PD | 2 |  |  |
|  | Schizotypal PD | 1 |  |  |
|  | OCPD | 1 |  |  |

*Note*. Significant group differences are indicated by **p*<.05, ***p*<.01, ****p*<.001.

**Table S2**
*Bivariate Spearman’s Rho correlations between DIDS, SCIM and PID-5-SF PDs*

|  |  | 1 | 2 | 3 | 4 | 5 | 6 | 7 | 8 | 9 | 10 | 11 | 12 | 13 | 14 |
| --- | --- | --- | --- | --- | --- | --- | --- | --- | --- | --- | --- | --- | --- | --- | --- |
| DIDS | 1. Exploration in breadth | - | .44** | .06 | .50** | **.53**** | .40** | .16 | .03 | .07 | -.13 | .02 | .09 | -.03 | .03 |
|  | 2. Exploration in depth | **.59**** | - | .03 | .48** | .47** | .11 | .22 | .11 | -.01 | -.09 | .05 | .08 | .03 | .05 |
|  | 3. Ruminative exploration | .27* | .09 | - | -.33* | -.26 | -.20 | .33* | .39** | .32* | .44** | .40** | .06 | .34* | .38** |
|  | 4. Commitment making | .37** | **.52**** | -.31** | - | **.76**** | .34* | .09 | .03 | .06 | -.17 | .03 | .15 | .06 | .03 |
|  | 5. Identification with commitment | .36** | .45** | -.34** | **.73**** | - | .32* | .05 | -.02 | -.05 | -.15 | -.11 | .06 | .09 | .03 |
| SCIM | 6. Consolidated identity | .16 | .12 | -.18 | .20 | .27* | - | .09 | .08 | .10 | -.05 | .07 | .21 | .08 | .09 |
|  | 7. Disturbed identity | .25* | .09 | .33** | -.06 | -.21 | -.15 | - | **.59**** | **.55**** | .37** | **.55**** | **.57**** | .45** | **.55**** |
|  | 8. Lack of identity | .20 | .00 | .48** | -.15 | -.21 | -.25* | **.64**** | - | **.57**** | **.62**** | **.73**** | .43** | **.52**** | **.64**** |
| PDs | 9. Antisocial PD | .15 | .11 | .22* | .01 | -.09 | -.04 | **.51**** | .24 | - | **.57**** | **.86**** | **.72**** | **.64**** | **.76**** |
|  | 10. Avoidant PD | .02 | -.04 | .34** | -.12 | -.26* | -.17 | .39** | .42** | **.57**** | - | **.66**** | .46** | **.77**** | **.80**** |
|  | 11. Borderline PD | .06 | .07 | .31** | -.06 | -.22 | -.12 | **.55**** | .34** | **.85**** | **.67**** | - | **.62**** | **.60**** | **.73**** |
|  | 12. Narcissistic PD | .15 | .08 | .20 | -.05 | -.11 | -.03 | .47** | .24 | **.79**** | .49** | **.66**** | - | **.57**** | **.62**** |
|  | 13. Obsessive-compulsive PD | .03 | -.03 | .18 | -.04 | -.13 | -.03 | .43** | .30** | **.73**** | **.72**** | **.72**** | **.58**** | - | **.83**** |
|  | 14. Schizotypal PD | .15 | .01 | .30** | -.20 | -.16 | -.11 | .47** | .40** | **.72**** | **.76**** | **.73**** | **.59**** | **.82**** | - |

*Note*. Strong correlations (>.50; Cohen, 1988) are presented in bold, very strong correlations (>.70) are also underscored. The correlational values above the diagonal represent the patient sample and those below the diagonal represent the non-clinical control group. Bonferroni correction: for the correlational analyses between DIDS, SCIM and PID-5-SF PDs, the *p-*values were considered statistically significant at *p*<.008 (i.e., .05/6 PDs). **p*<.008, ***p*<.001.

**Table S3**
*Bivariate Spearman’s Rho correlations between DIDS, SCIM and PID-5-SF MPTs*

|  |  | 1 | 2 | 3 | 4 | 5 | 6 | 7 | 8 | 9 | 10 | 11 | 12 | 13 | 14 | 15 | 16 | 17 | 18 | 19 | 20 | 21 | 22 | 23 | 24 | 25 | 26 | 27 | 28 | 29 | 30 | 31 | 32 | 33 |
| --- | --- | --- | --- | --- | --- | --- | --- | --- | --- | --- | --- | --- | --- | --- | --- | --- | --- | --- | --- | --- | --- | --- | --- | --- | --- | --- | --- | --- | --- | --- | --- | --- | --- | --- |
| DIDS | 1. Exploration in breadth | - | .44** | .06 | .50** | **.53**** | .40** | .16 | .03 | .05 | -.08 | .10 | .12 | .13 | .03 | -.16 | -.19 | -.11 | -.06 | -.14 | -.08 | .05 | -.05 | .02 | -.02 | .12 | -.01 | .06 | -.04 | .09 | .11 | .01 | .07 | .09 |
|  | 2. Exploration in depth | **.59**** | - | .03 | .48** | .47** | .11 | .22 | .11 | .07 | -.17 | -.07 | .07 | .07 | .02 | -.09 | -.08 | -.06 | -.06 | -.18 | .03 | .09 | -.07 | .09 | .16 | .19 | .01 | .05 | -.01 | .05 | .24 | .05 | .05 | .12 |
|  | 3. Ruminative exploration | .27* | .09 | - | -.33** | -.26 | -.20 | .33* | .39** | -.06 | .22 | .19 | .26 | .43** | .13 | .36** | .35** | .26 | .25 | .28 | .39** | .23 | .32* | .22 | .21 | .23 | .22 | .29 | .34* | .24 | .15 | .32* | .33** | .29 |
|  | 4. Commitment making | .37** | **.52**** | -.31** | - | **.76**** | .34* | .09 | .03 | .17 | -.08 | .09 | .03 | -.01 | .09 | -.24 | -.25 | -.07 | -.10 | -.16 | -.08 | .08 | .04 | .09 | -.09 | .19 | -.03 | .09 | -.08 | .10 | .32* | .03 | .10 | .04 |
|  | 5. Identification with com. | .36** | .45** | -.34** | **.73**** | - | .32* | .05 | -.02 | .04 | -.12 | -.02 | .07 | -.07 | -.06 | -.22 | -.22 | -.02 | -.01 | -.12 | -.16 | -.04 | -.06 | -.06 | -.04 | .11 | -.10 | -.06 | -.10 | -.04 | .30 | -.01 | .04 | .04 |
| SCIM | 6. Consolidated identity | .16 | .12 | -.18 | .20 | .27* | - | .09 | .08 | .22 | -.02 | .11 | .16 | .06 | .09 | -.09 | -.06 | .02 | .07 | -.07 | .01 | .09 | .06 | .05 | .09 | .18 | .05 | .14 | .12 | .19 | .16 | .17 | .11 | .06 |
|  | 7. Disturbed identity | .25* | .09 | .33** | -.06 | -.21 | -.15 | - | **.59**** | .49** | .36** | **.51**** | **.52**** | **.61**** | .37** | .32* | .37** | .33* | .28 | .14 | .38** | .50** | .47** | .36** | .45** | .41** | **.52**** | .48** | .49** | .44** | .26 | **.52**** | **.57**** | **.52**** |
|  | 8. Lack of identity | .20 | .00 | .48** | -.15 | -.21 | -.25* | **.64**** | - | .31 | .32* | .40** | .47** | **.62**** | .35* | **.60**** | **.60**** | .37** | .30 | .37** | **.61**** | **.60**** | **.52**** | **.54**** | .30 | **.57**** | .49** | .57** | .42** | **.56**** | .39** | **.59**** | **.56**** | **.53**** |
| MPTs | 9. Attention seeking | .15 | .10 | .17 | -.02 | -.09 | -.02 | .43** | .19 | - | .32* | **.57**** | .43** | .48** | **.60**** | .14 | .23 | .17 | .15 | .10 | .35* | **.57**** | .49** | .49** | .24 | .31* | **.56**** | .30 | .48** | .49** | .42** | .46** | .39** | .40** |
|  | 10. Callousness | .10 | -.09 | .12 | -.05 | -.04 | .02 | .23 | .27* | .35** | - | **.65**** | **.62**** | .49** | **.51**** | .42** | .44** | **.56**** | **.58**** | **.58**** | .35* | .48** | **.52**** | .29 | .34* | **.53**** | .46** | .40** | .47** | **.58**** | .37** | **.51**** | **.57**** | .50** |
|  | 11. Deceitfulness | .09 | .04 | .24 | -.04 | -.13 | -.11 | .50** | .35** | **.57**** | .46** | - | **.67**** | **.64**** | **.71**** | .33* | .43** | .29 | .38** | .33* | .42** | **.61**** | **.57**** | .44** | .27 | .44** | **.58**** | **.54**** | **.58**** | **.62**** | .36** | **.55**** | **.63**** | .48** |
|  | 12. Grandiosity | .13 | .01 | .20 | -.08 | -.12 | -.04 | .45** | .29* | **.60**** | .46** | **.58**** | - | **.71**** | **.55**** | **.57**** | .50** | **.56**** | **.57**** | .49** | **.52**** | .49** | **.53**** | .37** | .50** | **.64**** | **.51**** | .46** | **.56**** | **.51**** | **.53**** | **.63**** | **.68**** | **.64**** |
|  | 13. Hostility | .05 | -.04 | .28* | -.18 | -.22 | -.12 | .41** | .25 | .47** | .42** | **.54**** | **.74**** | - | **.53**** | .53** | **.58**** | .37** | .39** | .40** | **.68**** | **.73**** | **.68**** | **.57**** | .44** | **.65**** | **.67**** | **.69**** | **.61**** | **.75**** | .43** | **.65**** | **.62**** | **.63**** |
|  | 14. Manipulativeness | .16 | .27* | .16 | .10 | .03 | .01 | .37** | .10 | **.69**** | .34** | **.67**** | **.62**** | **.55**** | - | .35* | .35* | .21* | .34* | .20 | .43** | .47** | .50** | .41** | .26 | .47** | .43** | .43** | **.53**** | **.61**** | .41** | .48** | .49** | .44** |
|  | 15. Anhedonia | .02 | -.09 | .29** | -.13 | -.24 | -.10 | .29* | .35** | .33** | .39** | .45** | .40** | .38** | .33** | - | **.64**** | .50** | .45** | .62** | **.64**** | .45** | .38** | .36* | .40** | **.56**** | .40** | .48** | .33* | .44** | .36** | .38** | **.58**** | **.53**** |
|  | 16. Depressivity | .12 | 01 | .34** | -.03 | -.08 | -.13 | .26* | .43** | .20 | .47** | .33** | .30** | .31** | .22 | .48** | - | .44** | .38** | .46** | **.60**** | **.54**** | .40** | .41** | .42** | .49** | .46** | .41** | .46** | **.52**** | .29 | .47** | **.54**** | .47** |
|  | 17. Intimacy avoidance | -.03 | -.05 | .16 | -.16 | -.23 | -.11 | .24 | .25 | .16 | .31** | .26 | 33** | .29** | .24 | .37** | .41** | - | **.62**** | **.69**** | .32* | .33* | .36** | .13 | .39** | .46** | .23 | .25 | .30 | .25 | .39** | .40** | .50** | .43** |
|  | 18. Restricted affectivity | .05 | -.09 | .16 | -.09 | -.12 | -.02 | .31** | .30** | .38** | **.52**** | .49** | .49** | **.51**** | .43** | .40** | .33** | .42** | - | **.57**** | .27 | .17 | .40** | .19 | .37** | .43** | .33* | .31 | .41** | .35* | .39** | .42** | .40** | .35* |
|  | 19. Withdrawal | .04 | -.06 | .18 | -.04 | -.11 | -.08 | .28* | .36** | .25 | .49** | .36** | .46** | .46** | .31** | .45** | .40** | .45** | **.52**** | - | .40** | .35* | .42** | .20 | .28 | **.51**** | .29 | .26 | .26 | .36** | .43** | .42** | .42** | .41** |
|  | 20. Anxiousness | .06 | .08 | .40** | -.03 | -.16 | -.23 | .42** | .41** | .40** | .29* | .50** | .42** | **.53**** | .40** | .47** | .33** | .32** | .36** | .47** | - | **.66**** | **.55**** | **.64**** | .47** | **.65**** | **.52**** | .47** | .53** | .49** | .47** | .48** | **.58**** | **.59**** |
|  | 21. Emotional lability | .05 | -.11 | .24 | -.11 | -.23 | -.14 | .46** | .33** | .35** | .30** | .39** | .43** | .47** | .27* | .31** | .31** | .34** | .23 | .30** | .49** | - | **.68**** | **.61**** | .34* | **.59**** | **.72**** | **.63**** | **.56**** | **.69**** | .45** | **.63**** | **.59**** | .57 |
|  | 22. Perseveration | .01 | -.01 | .15 | -.07 | -.07 | .02 | .41** | .21 | .43** | .45** | **.57**** | .50** | **.56**** | .49** | .35** | .25 | .44** | **.52**** | .37** | .45** | **.51**** | - | **.59**** | .50** | .49** | **.61**** | **.61**** | **.54**** | **.61**** | **.56**** | **.73**** | .50** | .49** |
|  | 23. Separation insecurity | .03 | .13 | .16 | -.01 | -.18 | -.03 | .48** | .29** | .42** | .12 | .44** | .30** | .33** | .43** | .28* | .23 | .19 | .22 | .22 | .43** | .44** | .42** | - | .31 | .49** | **.60**** | **.54**** | .47** | .47** | .42** | **.55**** | .42** | .49** |
|  | 24. Submissiveness | -.01 | -.06 | .16 | -.05 | -.17 | -.07 | .39** | .29** | .37** | .27* | .39** | .37** | .32** | .39** | .42** | .27* | .28* | .37** | .33** | .48** | .43** | .47** | .40** | - | .48** | .34* | .37** | .41** | .23 | .43** | **.51**** | .38** | .41** |
|  | 25. Suspiciousness | .07 | .08 | .24 | -.04 | -.24 | -.09 | .39** | .30** | .22 | .37** | .36** | .44** | .50** | .33** | .34** | .41** | .40** | .38** | .37** | .46** | .47** | .44** | .48** | .34** | - | .44** | **.53**** | .42** | **.59**** | **.65**** | **.59**** | **.57**** | **.58**** |
|  | 26. Distractibility | .14 | .09 | .14 | -.02 | -.14 | .06 | .41** | .21 | **.51**** | .26 | **.54**** | .48** | .44** | **.52**** | .41** | .27* | .35** | .41** | .27* | .41** | .44** | **.58**** | .47** | .41** | .29* | - | **.59**** | **.56**** | **.60**** | .41** | **.57**** | .50** | **.55**** |
|  | 27. Impulsivity | .03 | -.03 | .20 | -.08 | -.20 | -.04 | .33** | .06 | .48** | .37** | .40** | .39** | .37** | .39** | .29* | .16 | .26* | .31** | .24 | .40** | .39** | .50** | .32** | .46** | .33** | .35** | - | .40** | **.70**** | .30 | **.56**** | .48** | .46** |
|  | 28. Irresponsibility | .14 | -.03 | .22 | -.02 | -.10 | -.03 | .44** | .34** | .38** | .42** | **.57**** | .40** | .35** | .36** | .39** | .37** | .27* | .35** | .33** | .41** | .37** | .39** | .34** | .33** | .29** | **.52**** | .23 | - | **.53**** | .36* | **.60**** | **.60**** | .48** |
|  | 29. Risk taking | .11 | .16 | .13 | .09 | -.04 | -.03 | .29* | .06 | .45** | .35** | .49** | .47** | .43** | **.58**** | .18 | .22 | .27* | .36** | .23 | .24 | .29** | **.51**** | .37** | .28* | .32** | .43** | **.51**** | .36** | - | .36* | **.62**** | **.55**** | **.53**** |
|  | 30. Rigid perfectionism | .03 | .01 | .09 | .07 | -.05 | -.05 | .31** | .16 | .44** | .28* | .48** | .48** | .47** | .49** | .17 | .15 | .30** | .34** | .36** | .48** | .42** | .50** | **.54**** | .30** | .41** | .44** | .35** | .19 | .41** | - | .44** | .38** | .45** |
|  | 31. Eccentricity | .18 | .04 | .30* | -.00 | -.05 | -.20 | .37** | .30** | .36** | .43** | .44** | .50** | .46** | .32** | .39** | .47** | .45** | .38** | .46** | **.53**** | .45** | .49** | .34** | .32** | .40** | .47** | .47** | .45** | **.52**** | .45** | - | **.58**** | **.56**** |
|  | 32. Perceptual dysregulation | .15 | .11 | .25 | .00 | -.07 | -.09 | .43** | .37** | .46** | .40** | .47** | **.57**** | .40** | .40** | .38** | **.51**** | .36** | .37** | .39** | .32** | .38** | .42** | .36** | .31** | .49** | .38** | .39** | .40** | .37** | .39** | **.59**** | - | **.80**** |
|  | 33. Unusual beliefs | .08 | -.03 | .30** | .03 | -.13 | -.20 | .35** | .28* | .28* | .38** | .43** | .42** | .32** | .36** | .30** | .39** | .35** | .32** | .34** | .35** | .41** | .34** | .39** | .22 | **.52**** | .36** | .24 | .35** | .36** | .44** | **.56**** | **.55**** | - |

*Note*. Strong correlations (>.50; Cohen, 1988) are presented in bold, very strong correlations (>.70) are also underscored. The correlational values above the diagonal represent the patient sample and those below the diagonal represent the non-clinical control group. Bonferroni correction: for the correlational analyses between DIDS, SCIM and PID-5-SF MPTs, the *p*-values were considered statistically significant at .002 (i.e., .05/25 MPTs). **p*<.002, ***p*<.001.
